# On the protein folding problem in 2D-triangular lattices

**DOI:** 10.1186/1748-7188-8-30

**Published:** 2013-11-26

**Authors:** Abu Sayed Md Sohidull Islam, Mohammad Sohel Rahman

**Affiliations:** 1 AℓEDA Group, Department of CSE, BUET, Dhaka 1000, Bangladesh

**Keywords:** Protein folding, Approximation ratio, Algorithms, HP model

## Abstract

In this paper, we present a novel approximation algorithm to solve the protein folding problem in HP model. Our algorithm is polynomial in terms of the length of the given HP string. The expected approximation ratio of our algorithm is $1-\frac{2\log n}{n-1}$ for *n* ≥ 6, where *n*^2^ is the total number of H’s in a given HP string. The expected approximation ratio tends to reach 1 for large values of *n*. Hence our algorithm is expected to perform very well for larger HP strings.

## Background

A long standing problem in Molecular Biology and Biochemistry is to determine the three dimensional structure of a protein given only the sequence of amino acid residues that compose protein chains. This problem is known as the Holy Grail of Computational Molecular Biology, also termed as “cracking the second half of the genetic code”. There exist a variety of models attempting to simplify the problem by abstracting only the “essential physical properties” of real proteins. In these models, the three-dimensional space is often represented by a lattice. Residues which are adjacent (i.e., covalently linked) in the primary sequence must be placed at adjacent points in the lattice.

In this paper, we consider the Hydrophobic-Polar Model, HP Model for short, introduced by Dill [1]. The HP model is based on the assumption that hydrophobicity is the dominant force in protein folding. This model simplifies a protein’s primary structure to a linear chain of beads. Each bead represents an amino acid, which can be one of two types: **H** (hydrophobic or nonpolar) or **P** (hydrophilic or polar). Conformations of proteins are embedded in either a two-dimensional or three-dimensional square/triangular/hexagonal lattice. A *conformation* of a protein is simply a self-avoiding walk along the lattice. The goal of the protein folding problem is to find a conformation of the protein sequence on the lattice such that the overall *energy* is minimized, for some reasonable definition of energy. Each amino acid in the chain is represented by occupying one lattice point, connected to its chain neighbour(s) on adjacent lattice points. An optimal embedding is one that maximizes the number of H-H contacts which are not adjacent in the amino acid chain. So, in effect, an input to the problem is a finite string over the alphabet (*H*, *P*)^+^. Often, in what follows, the input strings to our problem will be referred to as HP strings. For a more biological background and motivations the readers are referred to [1,2].

A number of approximation algorithms have been developed for the HP model on the 2D square lattice, 3D cubic lattice, triangular lattice and the face-cantered-cubic (FCC) lattice [3-5]. The first approximation algorithm developed for this problem on the square lattice by Hart and Istrail has an approximation ratio of 1/4 [3]. The approximation ratio for this problem was improved to 1/3 by Newman [4]. The algorithm presented in [3] can be generalized to an approximation algorithm for the problem on the 3D cubic lattice. In [6], a general method for protein folding on the HP model was presented by Hart and Istrail. This method can be applied to a large class of lattice models. Hart and Istrail [7] provided the first approximation algorithn for the problem on the side-chain model which can be applied to 2D square, 3D cubic lattices, and FCC lattices. The approximation ratio achieved by [7] remains the best ratio for an approximation algorithm in any 3D HP-models to date. In [7], the authors also illustrate the transformation of approximation algorithm from lattice models to off-lattice models. Another approximation algorithm, based on different geometric ideas was presented in [5]. Heun [8] presented a linear-time approximation algorithm for protein folding in the HP side chain model on extended cubic lattice having approximate ration 0.84. In [9], the authors presented an approximation algorithm with approximation ratio 0.17 that folds an arbitrary protein sequence in the 2D hexagonal lattice HP-model. Readers are referred to a survey of Istrail and Lam [10] for further reading.

In [11], the authors proposed a genetic algorithm for the protein folding problem in HP model in 2D square lattice. In [12,13], hybrid genetic algorithm was presented for HP model in 2D triangular lattice and 3D FCC lattice. The authors in [14] first proposed the *pull move set* for rectangular lattices, which is used in the HP model under a variety of local search methods. They also showed the completeness and reversibility of the pull move set for rectangular grid lattices. In [15], the authors extended the idea of *pull move set* in local-search approach for finding an optimal embedding in 2D triangular grid and the FCC lattice in 3D.

In this paper, we present an approximation algorithm for protein folding in 2D-triangular lattice. To the best of our knowledge the best approximation ratio for this problem was obtained by Agarwalla et al. [2], which is $\frac{6}{11}$. For our algorithm we do a probabilistic analysis and deduce that the expected approximation ratio of our algorithm is $1-\frac{2\log n}{n-1}$ for *n* ≥ 6, where *n*^2^ is the total number of H in a given HP string. Clearly our approximation ratio depends on *n*, which in turn depends on the number of H in the HP string. For large values of *n*, this ratio tends to reach 1. So it can be expected that our algorithm would provide very close to optimal results for large values of *n*.

The rest of the paper is organized as follows. In Section ‘Preliminaries’, we define some notations and notions. Section ‘Our approach’ describes our approach to solve the problem. In Section ‘Expected approximation ratio ratio’ we deduce the expected approximation ratio. We briefly conclude in Section ‘Conclusion’.

## Preliminaries

In this section, we present some notions and definitions (mostly in relation to the underlying lattice) that we need to explain our algorithm. In a triangular lattice, each lattice point has six neighbouring lattice points [2]. In the literature it is also called a hexagonal lattice. Note that, by definition, a lattice is infinite. However, in what follows, when we refer to a lattice we will refer to a finite part of it. This finite part of the lattice would essentially be a hexagon. We now define some notions related to a hexagon in the context of our approach. Note that a hexagon is said to be perfect (or regular) if it has six equal sides and six equal angles. Throughout the paper, when we refer to a hexagon we assume that the opposite sides of it are parallel having the same length. Also, when we consider a non-regular hexagon we assume that the sides of it can be grouped into two groups based on their length. In particular, two of its sides (that are parallel to each other) have a particular length, say *p* and the other four sides have a different length, say *m*. Clearly, when *p* = *m*, we have a regular hexagon. Following the above discussion, it would be useful to define the former couple of sides of the (non-regular) hexagon (i.e., that having a length of *p* each) as -sides and the latter four sides (i.e., that having a length of *m* each) as -sides.

**Figure 1:**
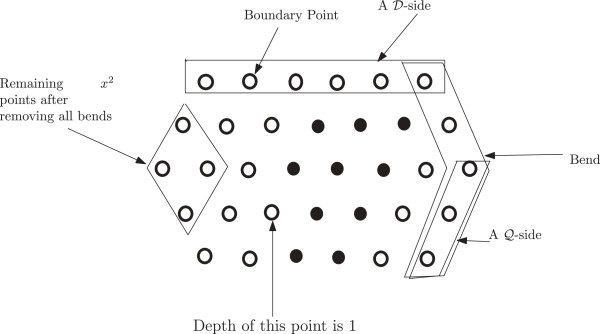
**Lattice.** In this figure, a lattice and some related notions are illustrated.

The discussion that follows can be better understood with the help of Figure 1. As has been mentioned above the finite portion of the lattice of our interest can be seen as a hexagon, the *boundary* of which consists of those lattice points that have fewer than six neighbours within the hexagon. An *edge* is formed by two neighbouring lattice points. If the lattice points are filled by H, the two neighbouring H’s also form an edge. If two H’s are non-adjacent in an HP string and placed on neighbouring lattice points to form an edge, they form a *bond*. The points on the boundary are referred to as the boundary points. The *depth* of a point in a lattice is the minimum number of points it needs to traverse to reach any boundary point. Naturally, the depth of a boundary point is 0. The depth of a hexagon is the maximum depth of all points in the hexagon. In Figure 1, the depth of the hexagon is 2.

The *length* of the hexagon (or lattice) is the total number of points along the -sides. Figure 1, shows a hexagon of length 6. A *region* in the hexagon is a group of the lattice points such that each point in it has at least two neighbours from within it. Similar to the boundary of a hexagon we also define the boundary of a region. The *boundary of a region* consists of those lattice points that have fewer than six neighbours within the region. A region must not contain any point such that deleting that point creates two separate regions. From a graph theoretic concept, the region cannot have a *cut vertex*. Also all the lattice points inside the boundary of a region are parts of the region. So, by definition, only the boundary itself cannot be considered as a region unless there are no points inside the boundary at all. In Figure 1, the black vertices comprise a region (which has only one point inside the boundary). The size of a region is the total number of lattice points inside it including the boundary points.

We also introduce a notion of a *bend* for a hexagon if it’s length is greater than it’s depth. A bend refers to the combined bent line along the 2 -sides to the right (a bend could be defined identically considering the two -sides to the left as well. However, for our purpose, we exclude that option from our definition). A bend is illustrated in Figure 1. Notice that if the depth of such a hexagon is *x*, then a bend contains 2*x* + 1 points. There is a total of *ℓ* bends in a hexagon, where *ℓ* is the length of the hexagon. Removing all bends from the hexagon leaves a total of *x*^2^ lattice points (see Figure 1).

**Figure 2:**
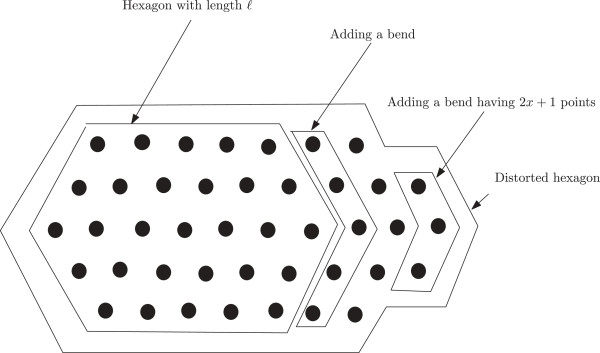
**Distorted hexagon.** In this figure, a distorted hexagon is illustrated.

Now we define a new notion of a distorted hexagon as follows. Suppose we have a hexagon having length *ℓ* and depth *x*; so each bend contains 2*x* + 1 points. Now we can increase its length to get a new hexagon of length *ℓ* + 1 by adding a new bend. Similarly by adding succesive bends we can continue to increase the length of a hexagon. If within such a process the last added bend has fewer than 2*x* + 1 points, then we refer to the hexagon as a distorted hexagon. An example of a distorted hexagon is shown in Figure 2.

We use the usual notion of a *run* in an HP string. In particular, a run in an HP string denotes the consecutive H’s or P’s. For example, in the HP string *HHHPPHHPHHHH*, we have a run of 3 H’s, followed by a run of 2 P’s and so on. Here the *run-length* of the first run of H (P) is 3 (2). We sometimes will use the term H-run (P-run) to indicate a run of H’s (P’s). The longest H-run (P-run) of a string denotes the run of H (P) which has the highest run-length among all the H-runs (P-runs) of the string. For the sake of conciseness, the HP strings shall often be represented as H’s and P’s with the corresponding run-lengths as their powers. So, the HP string S=HHHPPHHPHHHH will often be conveniently represented by $Ŝ=H^{3}P^{2}H^{2}P^{1}H^{4}$. Further, we use *S*(*i*), 1 ≤v*i* ≤ |*S*| to denote the *i*th character of the HP string . Similarly, Ŝ(j) denotes the *j*th run of . For example, Ŝ(1) refers to *H*^3^, Ŝ(2) refers to *P*^2^ and so on. We will use *SumH* as the sum of the run-lengths of all the H-runs of a given string . We end this section with a formal definition of the problem we handle in this paper.

### 

**Problem 1. ***Given an HP string*, *the problem is to place the HP string on a triangular lattice such that the total number of bonds are maximized.*

## Our approach

Our approach is a simple and intuitive one. Our idea is to identify the length and depth of a suitable hexagon and then try to put all the H’s of a particular H-run inside the hexagon and put the P’s of the following P-run (if any) outside that hexagon. The length and depth of the hexagon depend on SumH. The motivation here is to get the maximum number of bonds between H’s. Note carefully that if we can fully fill a hexagon with *z* lattice points with a single H-run and get a total of *k* edges, the number of total bonds will be *k* - *z* + 1. And if the number of H-run is *n*(*H*) then in this case the total number of bonds will be *k* - *z* + *n*(*H*). We illustrate the approach with an example below. In the figures throughout this paper the bonds and edges are not shown explicitly. A connection between 2 lattice points indicate the presence of 2 H’s that are adjacent in the input HP string.

### 

**Example 1. ***Suppose we have an HP string as follows:*

$$Ŝ=H^{6}P^{5}H^{2}P^{6}H^{4}P^{5}H^{6}P^{3}H^{2}P^{5}H^{4}PH^{7}P^{6}H^{2}P^{2}H^{4}.$$

**Figure 3:**
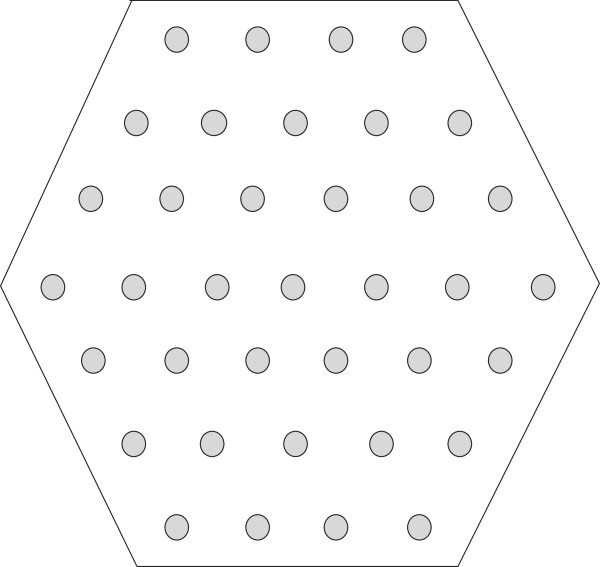
Lattice points, i.e., Hexagon.

**Figure 4:**
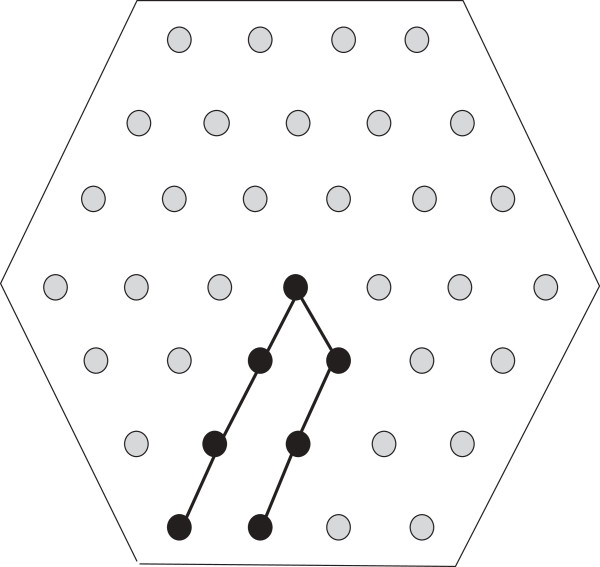
**Positioning**$\hat{S}(13)=H^{7}$**.**

**Figure 5:**
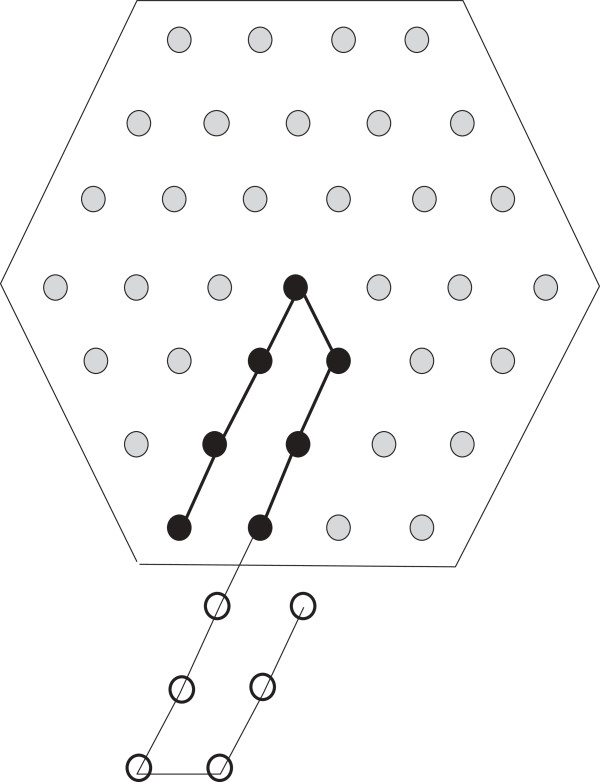
**Positioning**$\hat{S}(14)=P^{6}$**.**

**Figure 6:**
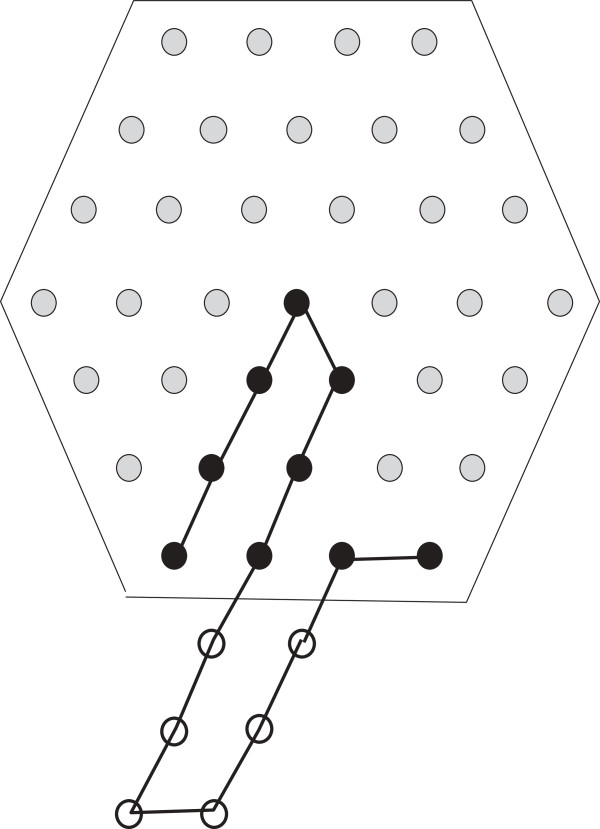
**Positioning**$\hat{S}(15)=H^{2}$**.**

**Figure 7:**
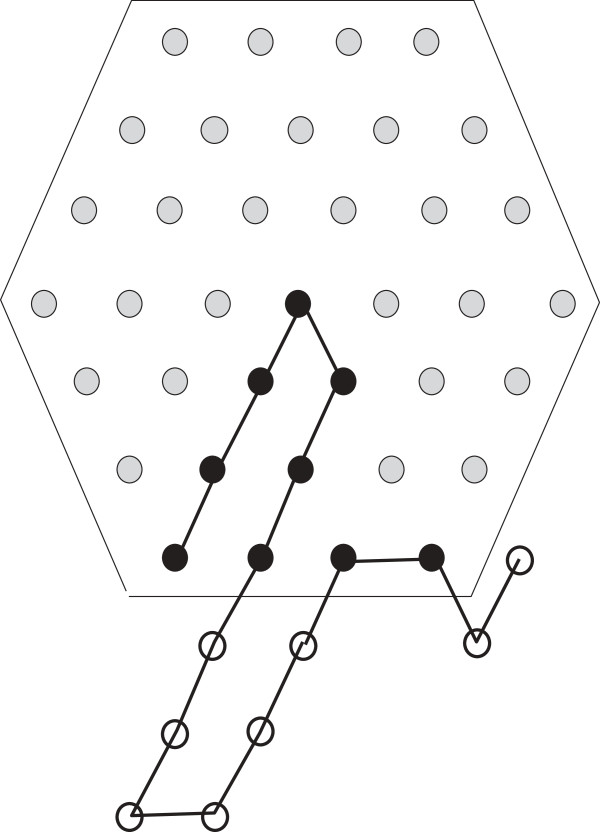
**Positioning**$\hat{S}(16)=P^{2}$**.**

**Figure 8:**
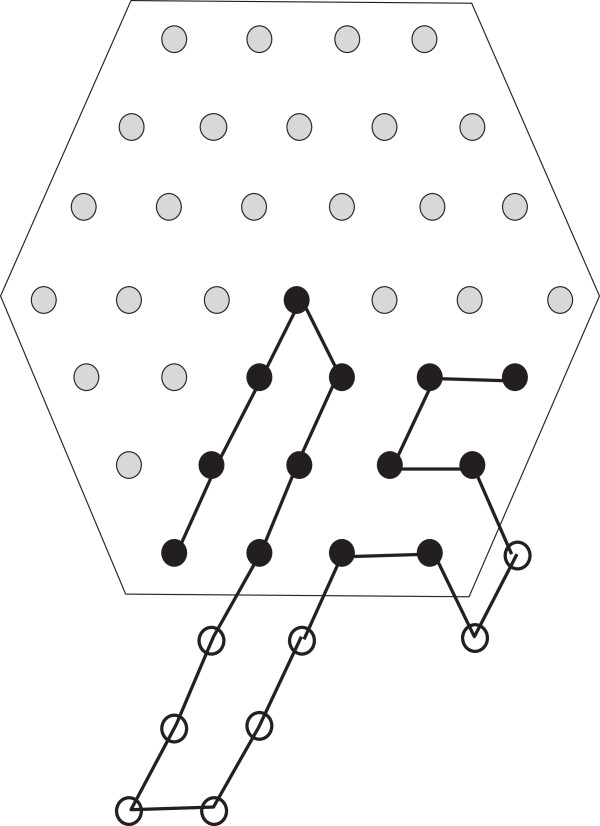
**Positioning**$\hat{S}(13)=H^{4}$**.**

**Figure 9:**
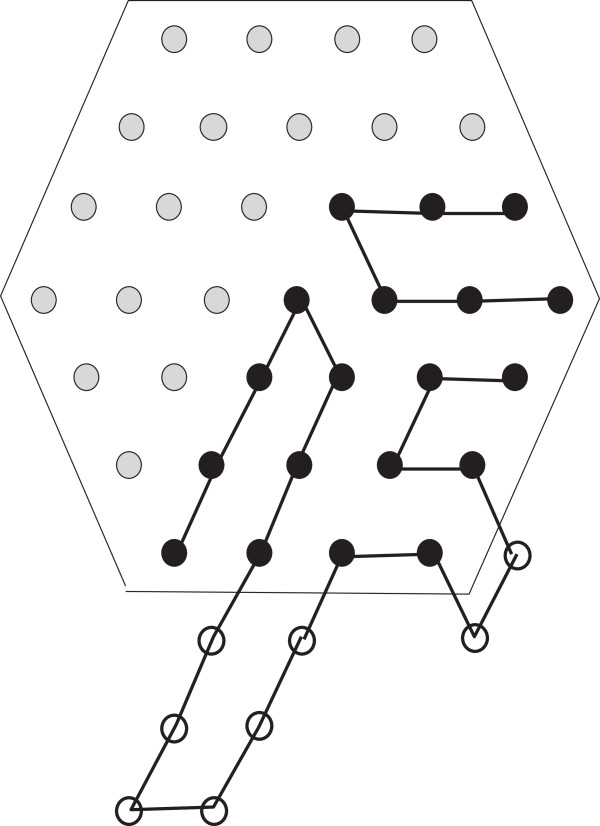
**Positioning**$\hat{S}(13)=H^{6}$**.**

**Figure 10:**
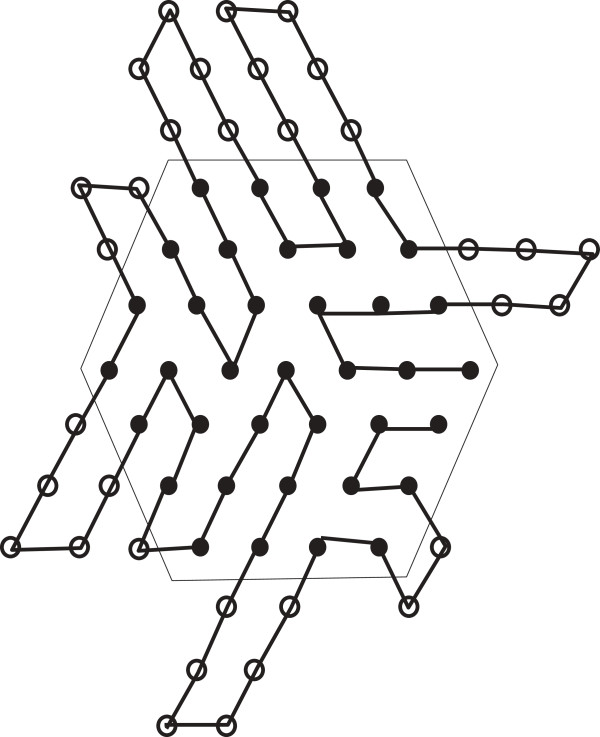
Final state.

*Figure*3*gives us a suitable regular hexagon for**on the underlying lattice. Our approach starts with the longest H-run of*. *In Figure*4*the longest H-run*, *i.e*, $Ŝ(13)=H^{7}$, *is first positioned within the hexagon*. *Then*, *in Figure*5, *the subsequent P-run is positioned outside the hexagon*. *Similarly the approach continues through Figures*6, 7 and 8*where we illustrate the positioning of H-runs and P-runs up to*Ŝ(17). *Then we wrap around and start with*Ŝ(1)*in Figure*9. *The final position of all the runs of**is shown in Figure*10.

In Figure 10, we have a hexagon of 37 points and after filling the hexagon fully we get a total of 90 edges. It is easy to verify that the total number of bonds are 62 which is equal to *k* - *z* + *n*(*H*). Notably, if two hexagons have the same number of lattice points and are filled up fully with H by a given HP string, the hexagon with higher number of total edges have the higher number of total bonds as the difference between the total number of the edges and that of bonds is a constant.

Now that we have discussed our main approach to fill up the hexagon, we can shift our focus to the question of whether we can accommodate all the H-runs of the input HP string within the current hexagon. Recall that our goal is to increase the number of edges (and hence the total number of bonds)as much as possible. We have the following useful lemmas.

### 

**Lemma 1. ***If two hexagons have the same number of lattice points then the hexagon with the higher depth will not have lesser number of edges.*

### Proof

**Figure 11:**
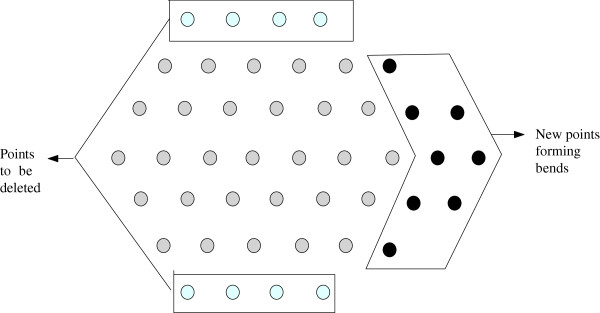
**Figure for Lemma 1.** This figure aids in understanding the proof of Lemma 1.

We show this by considering a hexagon and adjusting its depth keeping the total number of points fixed. We illustrate this scenario in Figure 11. Note that, the adjustment discussed and shown here does not give us a hexagon and is only to facilitate better exposition of the calculation and arguments.

Suppose that the total number of points is *z* and the depth of the hexagon *x* and length *ℓ*. If we remove the top and bottom rows and put the corresponding points along the other rows of the hexagon, new bends to the either side of the hexagon will be created and each bend will have 2 × (*x* - 1) + 1 points. The new hexagon will have depth *x* - 1 and additional$\frac{2\ell }{2x-1}\geq 1$ bends (see Figure 11).

Now deleting two rows decrease 2 × (2*ℓ* + *ℓ* - 1) or 6*ℓ* - 2 edges. On the other hand, increasing the$\frac{2\ell }{2x-1}$ bends will increase$\frac{2\ell }{2x-1}\times ((2x-2)+(2x-1)+(2x-2))$ or$\frac{2\ell }{2x-1}\times (6x-5)$ or$\frac{2\ell }{2x-1}\times (3(2x-1)-2)$ or$6\ell -\frac{4\ell }{2x-1}$ edges. This is clearly less than or equal to 6*ℓ* - 2 as$\frac{4\ell }{2x-1}-2\geq 0$ or$\frac{2\ell }{2x-1}\geq 1$. Hence the result follows. □

### 

**Lemma 2. ***Suppose we have a regular hexagon*$H_{1}$*containing N points*. *Now we reduce the length of this hexagon to get another hexagon*$H_{2}$*such that*$H_{2}$*contains N points as well*. *Then*$H_{2}$*will have lesser number of edges inside it than that of*$H_{1}$.

### 

**Figure 12:**
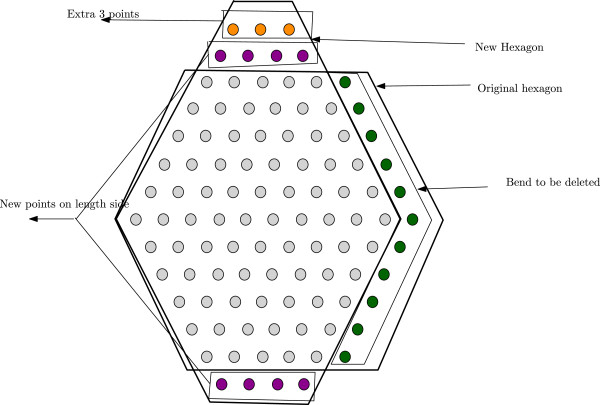
**Figure for Lemma 2.** This figure aids in understanding the proof of Lemma 2.

*Proof.* Suppose that the depth of the regular hexagon$H_{1}$ is *x*. So the length is *x* + 1. To reduce the length of$H_{1}$, while keeping the total number of points intact, we have to remove a bend of$H_{1}$ and distribute the points on that bend over the adjacent sides (See Figure 12). Hence$H_{2}$ will be a non-regular hexagon. Note that a bend in a regular hexagon contains 2*x* + 1 lattice points. After reducing the length, new length will become *x*-1 as follows. By deleting one bend we reduce the length of adjacent sides by one. So they will now have *x* lattices points. After placing the removed lattice points over these sides the new side will definitely have *x* - 1 lattice points.

Now deleting a bend decreases 2 × 2*x* + 1 + 2*x* or 6*x* + 1 edges. Out of the 2*x* + 1 points of the bend, 2*x* - 2 points will create new sides. These points create additional 2 × (2 × (*x* - 1) + *x* - 2) or 6*x* - 8 edges. And the rest of the 3 points can contribute to at most 8 edges. So we get at most a total of 6*x* edges whereas we loose 6*x* + 1 edges. Hence the result follows. □

### 

**Lemma 3. ***Assuming that we can fill up all the points of the hexagon*, *the total number of edges (as well as the total number of bonds) will be maximum if*, *and only if*, *the hexagon is a regular hexagon*.

### 

*Proof.* Lemma 3 follows readily from Lemmas 1 and 2. □

**Figure 13:**
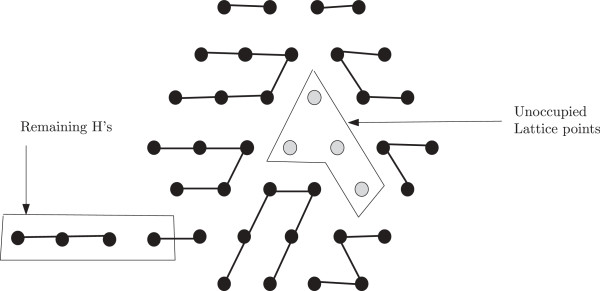
$\hat{S}$**of Example 2 cannot properly fill the hexagon.** This figure illustrates that$\hat{S}$ of Example 2 cannot properly fill the hexagon.

As has been mentioned before, our algorithm proceeds in an iterative fashion in order to achieve the highest possible number of edges by iteratively changing the length and depth of the hexagon. We start with an appropriate regular hexagon. Note carefully that, by Lemma 3, if we can fill the points of a regular hexagon, we get the optimum number of edges. If we fail to fill up all the points of a regular hexagon we put the rest of the H-runs outside the hexagon in a single row (see Figure 13) and finally compute the total number of bonds. We reduce the depth of the hexagon and increase its length with the hope that the number of bonds will increase in the new hexagon. We continue the iteration (i.e., reducing the depth of the hexagon and filling it up) until we reach a case when the total number of bonds decreases than that of the previous iteration. In that case, we terminate our algorithm and return the result of the previous iteration.

Notably, to fill up a regular hexagon with depth *x* at least one H-run having length 2*x* + 1 is needed. Besides, we need at least two H-runs of length 2*x*, three of length 2*x* - 2, three of length 2*x* - 4… three H-runs of length 1 or 2 (depending on the size of *x*) in the input. The string in Example 1 presented before meets this criteria assuming *x* = 3. To explain a bit more, note that, in the HP string of Example 1 we have one H-run with run-length 7, two H-runs with run-length 6, three H-runs with run length 4 and the rest of the H-runs have run-length 2. Another example is given below where we cannot put all H’s in a regular hexagon.

### 

**Example 2. ***Consider an HP string*

$$\;Ŝ=H^{6}P^{3}H^{4}P^{4}H^{3}P^{6}H^{4}P^{3}H^{2}P^{4}H^{2}P^{3}H^{6}P^{2}H^{5}P^{2}H^{2}PH^{3}.$$

**Figure 14:**
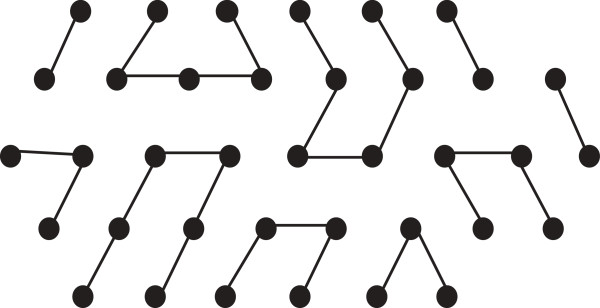
$\hat{S}$**of Example 2 can fill the adjusted hexagon.** This figure illustrates that$\hat{S}$ of Example 2 fill the adjusted hexagon.

*For this string*, *the length of a suitable regular hexagon is 4 (i.e.*, *depth is 3) as SumH is 37. But in Figure*13*we can see that we cannot properly fill the hexagon. So we put the rest of the H-runs outside the hexagon in a single row. In such a case we have to increase the length of the hexagon to 6 as well as decreasing its depth to 2. The new hexagon is shown in Figure*14. *It is evident from Figure*14, *that we can now fill the hexagon properly with**and thus increase the total number of bonds*.

### Steps of the algorithm

Now we describe the steps of our algorithm below. 

Step 1 Let SumH of the input HP string is *z* and the longest H-run isŜ(i) having run-length *k*. Compute$x=\lfloor \frac{1+\sqrt{1+\frac{4\times (z-1)}{3}}}{2}\rfloor$. Set *globalB* = 0.

Step 2 Set$\ell =\lfloor \frac{z-x^{2}}{2x+1}\rfloor$ and construct a hexagon with length *ℓ* and depth *x* where each bend will contain 2*x* + 1 points. If *z* ≥ *ℓ*(2*x* + 1) + *x*^2^ then add new *p* bends such that (*ℓ* + *p*)(2*x* + 1) + *x*^2^ ≥ *z* ≥ (*ℓ* + *p* + 1)(2*x* + 1) + *x*^2^. If *z* > (*ℓ* + *p*)(2*x* + 1) + *x*^2^ then create a distorted hexagon having the last bend (i.e., on the boundary of the hexagon) containing *z* - (*ℓ* + *p*)(2*x* + 1) + *x*^2^ points.

Step 3 For each of the H-runs and P-runs, starting fromŜ(i) and wrapping around the end (if applicable) execute the following steps. For an H-run, execute Step 3.a, Step 3.b and Step 3.c; for a P-run execute Step 3.d. 

Step a [for H-runs] If the run length of the H-run is less than 3 then we take lattice points on the boundary of the hexagon. Otherwise, we try to find a region from the remaining unoccupied points as follows. Here the total number of points in the region must be equal to the run-length of the current H-run and at least two of these points must be boundary points of the hexagon. We find the region executing the following steps (Steps 3.a.i to 3.a.iv). We ensure that the region property is maintained as we proceed by including the points one after another. In the following steps we will use to refer the the region we are constructing iteratively. 

Step i Take two points on the boundary of the hexagon. These are the first two points of the region.

Step ii Identify the unoccupied points in the hexagon such that each of those has two neighbouring lattice points in. Find the point having the highest depth among these points (breaking ties arbitrarily) and add this point to. Thus we increase the size (by one).

Step iii If no such point is found then go to Step 3.b.

Step iv If the size of is still less than the run length of the current H-run, go to Step 3.a.ii.

Step b [for H-runs]Fill up the lattice points of the identified region () with the H-run.

Step c [for H-runs]Put the rest of the H-runs (if any) outside the hexagon in a single row.

Step d [for P-runs]Put the P-run outside the hexagon in two rows. The first P of the run will be a neighbour of the previous H-run’s last H, while the last P of the run will be a neighbour of the next H-run’s first H.

Step 4 Count the total number of bonds, *B*.

Step 5 If *globalB* > *B*, return *globalB*.

Step 6 Otherwise set *globalB* = *B* and *x* = *x* - 1.

Step 7 If *x* = 1, return *B*; otherwise go to Step 2.

A brief discussion on Step 3.a.ii is in order. In Step 3.a.ii, our algorithm always chooses the points having the higher depths, with ties broken arbitralily. In some cases, some lattice points may remain unoccupied. Example 2 elaborates such cases. If some lattice points remain unoccupied we continue the algorithm. Some H-runs or part thereof may lie outside the hexagon (Step 3.c of the algorithm). We count the total number of bonds and compare it with previous/latter hexagon (increased or decreased depth) as applicable. So, if the algorithm fails to insert all the H-runs within a hexagon it does not mean it fails in total, as we put the rest of the H-runs or parts thereof (as appropriate) outside the hexagon. The algorithm is formally presented in Algorithm 1.

#### Algorithm 1 **Finding the Folding**

**Figure 15:**
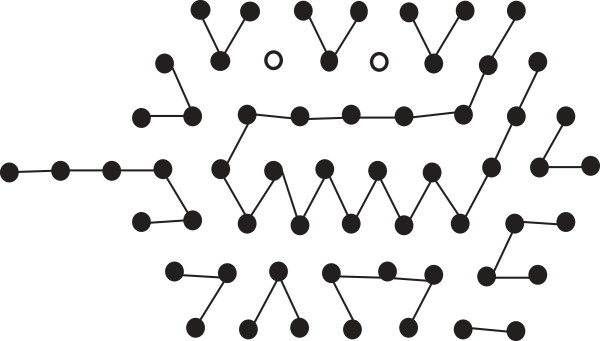
**Folding produced by Algorithm 1 with a hexagon havig depth 3 for**${\hat{S}}_{1}$**.** This figure illustrates the folding produced by by Algorithm 1 of${\hat{S}}_{1}$ of Section ‘Steps of the algorithm’ for hexagon having depth 3.

**Figure 16:**
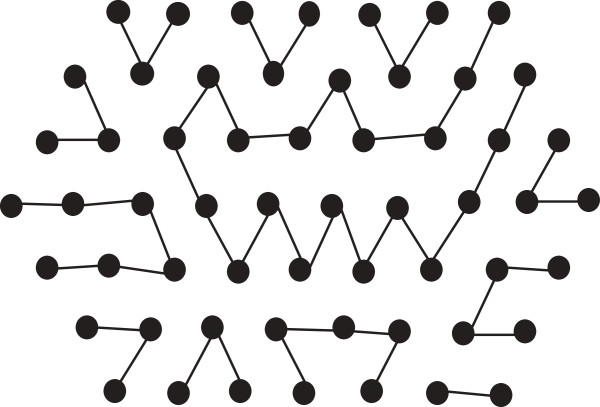
**Optimal folding for hexagon with depth 3 for**${\hat{S}}_{1}$**.** This figure illustrates the optimal filling of${\hat{S}}_{1}$ of Section ‘Steps of the algorithm’ for hexagon having depth 3.

**Figure 17:**
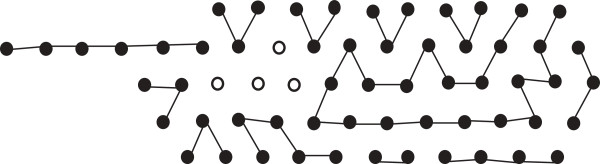
**Folding produced by Algorithm 1 with a hexgaon having depth 2 for**${\hat{S}}_{1}$**.** This figure illustrates the folding produced by by Algorithm 1 of$\hat{S}$ of Section ‘Steps of the algorithm’ for hexagon having depth 2.

In Figure 15 a folding produced by Algorithm 1 for the HP string${Ŝ}_{1}=H^{3}P^{3}H^{3}P^{4}H^{3}P^{6}H^{3}P^{3}H^{20}P^{4}H^{3}P^{3}H^{4}P^{2}H^{2}P^{2}H^{5}PH^{3}PH^{3}P^{2}H^{6}$ is shown for a hexagon with depth 3. The folding is not optimal for a hexagon with depth 3 as is evident from Figure 16, which shows the optimal folding. Now Algorithm 1 will count the total number of bonds and continue to the next iteration by reducing the depth by 1. The new folding produced in the next iteration is shown in Figure 17. Since the number of bonds in this folding is less than that of Figure 15, Algorithm 1 will choose the folding of Figure 15. So, as expected, Algorithm 1 may not produce the optimal folding for a hexagon with a given depth but it compares the folding produced by different hexagon having different depths and choose the best folding among there. As will be proved later, the folding produced by Algorithm 1 is expected to be quite near to optimal for long HP string. Now we present and prove the following theorem which basically proves the correctness of our approach.

#### 

**Theorem 1. ***Given a region consisting of lattice points, a starting and an ending points such that those are boundary points of the hexagon, there always exists a path that starts at the starting point, ends at the ending point visiting each point in the region exactly once.*

#### 

**Figure 18:**
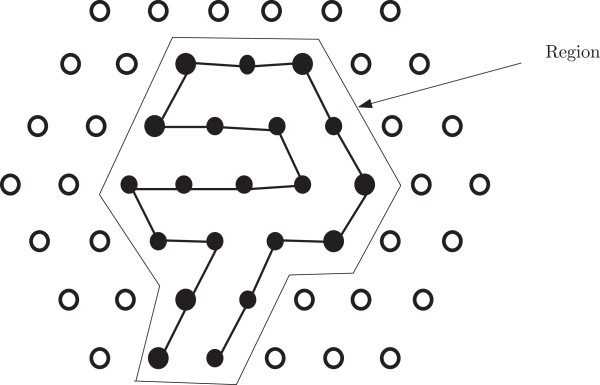
**For even number of rows.** This figure illustrates how to traverse all the points in a region with even number of rows.

**Figure 19:**
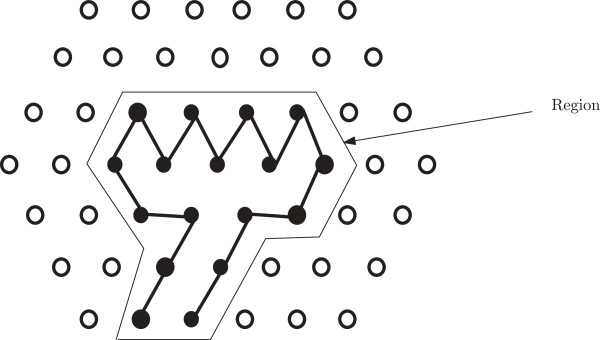
**For odd number of rows.** This figure illustrates how to traverse all the points in a region with odd number of rows.

*Proof.* We can traverse the points row wise from left to right within the region starting from, say, Row *i* and then right to left in Row *i* + 1 and so on. If the number of rows are even, then, in this manner we can traverse all the points (see Figure 18). If it is odd then we traverse in a similar way except for the last two rows, where we simultaneously traverse those in a zigzag fashion (see Figure 19). So filling up a region appropriately can be done in linear time with respect to the run-length of the corresponding H-run. □

Our algorithm runs in polynomial time as discussed below. Firstly, the algorithm iterates over at most *x* times. Now we have$x\leq \sqrt{z}$, because, *z* = 3 × *x* × (*x* + 1) which is proved in Lemma 4 in the following section. In each iteration we have to find a region for the current H-run. If a H-run has run-length *ℓ*, then Step 3.a in the algorithm needs *O*(*ℓ*^2^) time as Step 3.a.ii needs at most *O*(*ℓ*) time. So total time needed to perform this operation in each iteration, is at most *O*(*z*^2^). As each of the other steps need constant time, the complete runtime of the algorithm is$O(z^{2}\times \sqrt{z})$.

## Expected approximation ratio

In this section, we are going to deduce the expected approximation ratio of our algorithm. As the total number of H-runs and run-lengths thereof may vary, in this analysis, we will find the expected number of H-runs and the expected run-length of each of those. These two values will depend only on SumH. Consider a regular hexagon with depth *x*. Assume that the total number of points in the hexagon is *z*. Then we have the following lemma.

### 

**Lemma 4. ***Suppose we have a regular hexagon with depth x and z points. The total number of bonds*, *B*, *is* 6*x*^2 ^*when all the points are filled with H’s of a single H-run*.

### 

*Proof.* A regular hexagon with depth 1 have 1+6=7 points. We can increase its depth from *x* - 1 to *x* by adding 6*x* new points each having depth 0. Since we have *z* points in the hexagon, so we must have:

$$\begin{array}{lll} \;z & =1+6\times 1+6\times 2+6\times 3+\ldots +6\times x\; & \;\\ \Rightarrow z & =1+6\times (1+2+3+\ldots +x)\; & \;\\ \Rightarrow z & =1+6\times (x+1)x/2\; & \;\\ \Rightarrow z & =1+3\times x(x+1)\; & \; \end{array}$$

Now we will count the total number of possible edges, *E*. Note that each of the points except those in the perimeter can contribute to six edges. Among the points on the perimeter, the six corner points can only contribute to three edges whereas the others can contribute to four edges. Since each edge is formed by two points, to prevent double counting, we have to divide the total count by two. So we have the following:

$$\begin{array}{ll} \;2\times E & =6\times (3\times x\times (x+1)+1)-6\times 3-6\times 2\\ & \;\times (x-1)\\ \;\Rightarrow 2\times E & =2\times 3\times (3\times x\times (x+1)+1)-9+6-6x\\ \;\Rightarrow E & =3\times (3\times x\times (x+1)+1)-3-6x. \end{array}$$

Now we focus on calculating the total number of bonds *B*. Recall that according to our approach, only H’s are placed inside the hexagon. Since an H can have at most 2 H’s adjacent to it in an HP string, once placed inside the hexagon an H can only have at most 2 edges that would not be counted as bonds. So to compute *B* we simply need to deduct the total number of points from *E*. So we have:

$$\begin{array}{ll} \;B & =E-z+1\\ \Rightarrow B & =3\times (3\times x(x+1)+1)-3-6x-(3\times x(x+1)\\ & \;+1)+1\\ \Rightarrow B & =2\times (3\times x(x+1)+1)-3-6x+1\\ \Rightarrow B & =6\times x(x+1)+2-3-6x+1\\ \Rightarrow B & =6x^{2} \end{array}$$

This completes the proof. □

Now the following lemma considers non-regular hexagons as well.

### 

**Lemma 5. ***Consider a hexagon (either regular or non regular) having n*^2 ^*points. Then, the total number of bonds B is less than or equal *2 × *n*(*n* - 1).

### 

*Proof.* According to lemma 4 for a regular hexagon with 1 + 3 × *x* × (*x* + 1) points, the total number of bonds is 6*x*^2^. Or replacing *n* = *x* + 1 we get, for a regular hexagon with 1 + 3 × *n* × (*n* - 1) points, the total number of bonds is 6 × (*n* - 1)^2^. So, clearly we have:

$$\begin{array}{lll} \;B & \leq n^{2}\times (6\times {\left(n-1\right)}^{2})/(3\times n(n-1)+1)\; & \;\\ \Rightarrow B & \leq n^{2}\times (6\times {\left(n-1\right)}^{2})/(3\times n(n-1))\; & \;\\ \Rightarrow B & \leq 2\times n\times (n-1)\; & \; \end{array}$$

Hence the result follows for regular hexagons. Clearly, by Lemma 3 the result applies for non-regular hexagons as well. □

We will now deduce the approximation ratio based on an expected value of the total number of bonds. We assume that all H-runs have equal length. This assumption is valid in the context of our analysis and does not lose generality as follows. In what follows, we will be working with the expected number of H-runs and the expected length (say *k*_*Ex*_) of an H-run. Hence in our analysis, each H-run will be assumed to have length *k*_*Ex*_. We will now compute the expected values of the total number of edges (bonds), *E*_*Ex*_ (*B*_*Ex*_) under this assumption.

From Figure 1, we can see that, the length of the hexagon is *ℓ* and depth is *x*. So each bend contains 2*x* + 1 points and there are a total of *ℓ* bends. There are *x*^2^ remaining lattice points outside the *ℓ* bends. So if the total number of points are *z* (see Figure 1) then,

(1) $$z=(2x+1)\times \ell +x^{2}$$

So for a given *z* and *x* we can get,

(2) $$\ell =\frac{\left(z-x^{2}\right)}{2x+1}$$

To calculate the total number of edges, at first we have to identify how many edges can be formed by individual points. The arguments of Lemma 4 for calculating *E* and *B* also apply here. Note that, on the perimeter, aside from the corner points, total number of points are 2 × (*ℓ* - 2) + 4 × (*x* - 1). So *E*_*Ex*_ can be computed as follows:

$$\;\begin{array}{ll} 2E_{\text{Ex}} & =6\times ((2x+1)\times \ell +x^{2})-6\times 3-2\\ & \;\times (2\times (\ell -2)+4\times (x-1))\\ \Rightarrow 2E_{\text{Ex}} & =2\times 3\times ((2x+1)\times \ell +x^{2})-9\\ & \;-(2\ell -4+4x-4)\\ \Rightarrow E_{\text{Ex}} & =3\times ((2x+1)\times \ell +x^{2})-1-2\times (\ell +2x) \end{array}$$

And *B*_*Ex*_ can be computed as follows:

$$\begin{array}{ll} B_{\text{Ex}} & =E_{\text{Ex}}-z\\ B_{\text{Ex}} & =3\times ((2x+1)\times \ell +x^{2})-1-2\times (\ell +2x)\\ & \;-((2x+1)\times \ell +x^{2})\\ \Rightarrow B_{\text{Ex}} & =2\times ((2x+1)\times \ell +x^{2})-2\times (\ell +2x)-1 \end{array}$$

Hence, we get the following equation.

(3) $$B_{\text{Ex}}=2z-2\times (\ell +2x)-1$$

Note that according to our approach, the value of *x* is dependent on SumH. For this analysis, we now derive the expected run-length of H for a given HP string where SumH is *n*^2^. This problem can be mapped into the problem of *Integer Partitioning* as defined below.

### 

**Problem 2. ***Given an integer Y*, *the problem of Integer Partitioning aims to provide all possible ways of writing Y*, *as a sum of positive integers*.

Note that the ways that differ only in the order of their summands are considered to be the same partition. A summand in a partition is called a part. Now, if we consider SumH as the input of Problem 2 (i.e., *Y*) then each run-length can be viewed as parts of the partition. So at first, we have to find the expected number of partitions, i.e., the expected number of runs of H. Kessler and Livingston [16] showed that to get an integer partition of an integer *Y*, expected number of required parts is:

$$\sqrt{\frac{3Y}{2\pi }}\times (\log Y+2\gamma -2\log\sqrt{\frac{\pi }{6}}),$$

where *γ* is the famous Euler’s constant.

For our problem *Y* = *SumH* = *n*^2^. If we denote *E*[ *P*] as the expected number of H-runs then,

$$E[\;P]=\sqrt{\frac{6}{\pi }}\times n\times (\log n+\gamma -\log\sqrt{\frac{\pi }{6}}).$$

Now, as$\left(\log n+\gamma -\log\sqrt{\frac{\pi }{6}})\leq (\sqrt{\frac{2\pi }{3}}\times \log n\right)$ for *n* ≥ 5, we can say that

E[P]≤2n×logn.

Since SumH is *n*^2^, expected value of each part, i.e., expected length of each run is greater than or equal to$\frac{n^{2}}{2n\times \log n}=\frac{n}{2\log n}$. Since all the H-runs are assumed to have the same length so each of them will construct a bend of 2*x* + 1 points in the lattice. So we must have$2x+1\geq \frac{n}{2\log n}$. Hence we get the following equations:

(4) $$x\geq \frac{n}{4\log n}-\frac{1}{2}$$

(5) $$\ell \leq \frac{n^{2}-(\frac{n^{2}}{16{\left(\log n\right)}^{2}}-\frac{n}{4\log n}+\frac{1}{4})}{\frac{n}{2\log n}}$$

Now, let us consider a hexagon$H_{1}$ with length$\ell_{\text{max}}=\frac{n^{2}-(\frac{n^{2}}{16{\left(\log n\right)}^{2}}-\frac{n}{4\log n}+\frac{1}{4})}{\frac{n}{2\log n}}$ and depth$x_{\text{min}}=\frac{n}{4\log n}-\frac{1}{2}$. Now, in$H_{1}$ we also must have *n*^2^ points. So, from Lemma 1 and Equations 4 and 5, clearly the number of bonds in$H_{1}$ is less than or equal to than that in the hexagon having length *ℓ* and depth *x*. So from Equation 3 we have the following:

$$\begin{array}{ll} \;B_{\text{Ex}} & \geq 2n^{2}-2\times (2n\log n-\frac{n}{8\log n}-\frac{\log n}{2n}+\frac{1}{2}\\ & \;+\frac{n}{2\log n}-1)-1\\ \Rightarrow B_{\text{Ex}} & \geq 2n^{2}-2\times (2n\log n+\frac{3n}{8\log n}-\frac{\log n}{2n}) \end{array}$$

Now from Lemma 5, recall the upper bound for the total number of bonds, which is as follows: *B* ≤ 2 × *n*(*n* - 1). Hence we get the following expected approximation ratio:

$$\begin{array}{lll} \;\frac{B_{\text{Ex}}}{B} & \geq \frac{2n^{2}-2\times (2n\log n+\frac{3n}{8\log n}-\frac{\log n}{2n})}{2n\times (n-1)}\; & \;\\ \Rightarrow \frac{B_{\text{Ex}}}{B} & \geq \frac{n-2\log n-\frac{3}{8\log n}+\frac{\log n}{2n^{2}}}{n-1}\; & \; \end{array}$$

As the term$\frac{\log n}{2n^{2}}$ is very small we can ignore it from the final result. Hence we have:

$$\frac{B_{\text{Ex}}}{B}\geq \frac{n-2\log n-\frac{3}{8\log n}}{n-1}$$

As,$\frac{3}{8\log n}\leq 1$ for *n* ≥ 2, so,$\frac{B_{\text{Ex}}}{B}\geq \frac{n-2\log n-1}{n-1}$ or

$$\frac{B_{\text{Ex}}}{B}\geq 1-\frac{2\log n}{n-1}\;\text{for}\;n\geq 6.$$

This is the final expected approximation ratio.

Note that the ratio increases significantly with the increase of the value of *n* as presented in Table 1. So we can see that for large values of *n*, expected approximation ratio tends to 1. So for large *n* it is expected that our algorithm will outperform the approximation algorithm presented in [2]. Recall that the approximation ratio of the algorithm of [2] is$\frac{6}{11}$, i.e., around 0.55.

**Table 1:** **Expected approximation ratio for different values of *n***

**log *n***	***n***	**z *= n***^***2***^	**Ratio**
3	8	64	0.142
4	16	256	0.466
5	32	1024	0.677
6	64	4096	0.809

This table illustrates the expected approximation ratio for different values of *n*.

## Conclusion

In this paper, we have given a novel approximation algorithm to solve the protein folding problem in HP model introduced by Dill [1]. Our algorithm is polynomial and the expected approximation ratio is$1-\frac{2\log n}{n-1}$ for *n* ≥ 6 where *n*^2^ is total number of H in a given HP string. For larger HP strings it is expected that our algorithm will give better result than the algorithm provided in [2], which currently gives the best approximation ratio for 2D-triangular lattice. Additionally, our expected approximation ratio tends to reach one for large values of *n*. Hence our algorithm is expected to perform very well for larger HP strings.

## Competing interests

The authors declare that they have no competing interests.

## Authors’ contributions

ASMSI proposed the algorithms. MSR verified the correctness of the algorithm and analysis. Both the authors read and approved the manuscript.
